# Role of FGF and Hyaluronan in Choroidal Neovascularization in Sorsby Fundus Dystrophy

**DOI:** 10.3390/cells9030608

**Published:** 2020-03-04

**Authors:** Alyson Wolk, Dilara Hatipoglu, Alecia Cutler, Mariya Ali, Lestella Bell, Jian Hua Qi, Rupesh Singh, Julia Batoki, Laura Karle, Vera L. Bonilha, Oliver Wessely, Heidi Stoehr, Vincent Hascall, Bela Anand-Apte

**Affiliations:** 1 Cole Eye Institute & Lerner Research Institute, Cleveland Clinic Foundation, Cleveland, OH 44195, USA; wolka@ccf.org (A.W.); Hatipod@ccf.org (D.H.); cutlera@ccf.org (A.C.); alim2@ccf.org (M.A.); belll3@ccf.org (L.B.); qij@ccf.org (J.H.Q.); Singhr4@ccf.org (R.S.); Batokij@ccf.org (J.B.); lauraikarle@gmail.com (L.K.); bonilhav@ccf.org (V.L.B.); 2 Cleveland Clinic Lerner College of Medicine, Department of Molecular Medicine, Case Western Reserve University, Cleveland, OH 44195, USA; Wesselo@ccf.org; 3 Cleveland Clinic Lerner College of Medicine at Case Western Reserve University, Department of Ophthalmology, Cleveland, OH 44195, USA; 4 Department of Cardiovascular and Metabolic Sciences, Lerner Research Institute, Cleveland Clinic Foundation, Cleveland, OH 44195, USA; 5 Institute of Human Genetics, University of Regensburg, 93053 Regensburg, Germany; Heidi.Stoehr@klinik.uni-regensburg.de; 6 Department of Biomedical Engineering, Lerner Research Institute, Cleveland Clinic Foundation, Cleveland, OH 44195, USA; Hascalv@ccf.org

**Keywords:** sorsby’s fundus dystrophy, hyaluronan, neovascularization, retina

## Abstract

Sorsby’s fundus dystrophy (SFD) is an inherited blinding disorder caused by mutations in the tissue inhibitor of metalloproteinase-3 (*TIMP3*) gene. The SFD pathology of macular degeneration with subretinal deposits and choroidal neovascularization (CNV) closely resembles that of the more common age-related macular degeneration (AMD). The objective of this study was to gain further insight into the molecular mechanism(s) by which mutant TIMP3 induces CNV. In this study we demonstrate that hyaluronan (HA), a large glycosaminoglycan, is elevated in the plasma and retinal pigment epithelium (RPE)/choroid of patients with AMD. Mice carrying the S179C-TIMP3 mutation also showed increased plasma levels of HA as well as accumulation of HA around the RPE in the retina. Human RPE cells expressing the *S179C-TIMP3* mutation accumulated HA apically, intracellularly and basally when cultured long-term compared with cells expressing wildtype *TIMP3*. We recently reported that RPE cells carrying the *S179C-TIMP3* mutation have the propensity to induce angiogenesis via basic fibroblast growth factor (FGF-2). We now demonstrate that FGF-2 induces accumulation of HA in RPE cells. These results suggest that the TIMP3-MMP-FGF-2-HA axis may have an important role in the pathogenesis of CNV in SFD and possibly AMD.

## 1. Introduction

Sorsby’s fundus dystrophy (SFD) is a dominantly inherited, degenerative disease of the macula that is characterized by bilateral loss of central vision as a consequence of choroidal neovascularization (CNV) [1,2,3,4,5,6]. Specific mutations in the tissue inhibitor of metalloproteinase 3 (*TIMP3*) gene involving exon 5, exon 1 or the intron 4-exon 5 boundary have been shown to be causative [7,8,9,10,11,12,13,14]. In comparative studies using TIMP3 deficient mice, S179C-TIMP3 transgenic mice and in vitro culture experiments we have determined that TIMP3 partially inhibits angiogenesis by blocking the binding of vascular endothelial growth factor (VEGF) to VEGF Receptor 2 (VEGFR2). We have also demonstrated that the S179C-TIMP3 mutant protein induces angiogenesis via VEGF and fibroblast growth factor 2 (FGF-2) [15,16,17,18,19,20,21].

TIMP3 is produced constitutively by the retinal pigment epithelium (RPE) and choroidal endothelial cells [2,20]. It is a normal component of Bruch’s membrane [22] and binds to sulfated glycosaminoglycans of the extracellular matrix (ECM) [23,24]. Hyaluronan (HA) is a large glycosaminoglycan that is a significant component of peri-cellular and extracellular matrices. HA is essential for numerous physiological functions that are dependent on its chain size and its interactions with various effector proteins and receptors [25]. HA has been implicated in the regulation of neovascularization and endothelial barrier function [26]. While studies have demonstrated that signaling via HA and its cell surface receptor CD44 accentuates CNV in mice using a laser-induced model [27], the exact molecular mechanism by which HA regulates tissue remodeling and neovascularization is unknown.

We have recently reported that RPE cells expressing mutant TIMP3 secrete increased amounts of FGF-2 [28] and that this contributes to increased angiogenesis. FGF-2 has been shown to be important in tumor angiogenesis, but its role in CNV has been less well studied. The most direct evidence for a role of FGF-2 in CNV comes from studies in which Flk1-Cre or Tie2-Cre mediated deletions of FGF receptor 1 (FGFR1) and FGF receptor 2 (FGFR2) in endothelial cells resulted in reduced laser-induced CNV in mice [29]. Extracellular matrix components such as heparan sulfate proteoglycans (HSPGs) bind and regulate the activity of growth factors such as FGF-2 [30] and have a critical role in the regulation of neovascularization [31]. In addition, the observation that activation of the FGFR-STAT3 pathway can induce a hyaluronan-rich microenvironment that can affect tumor growth [32] led us to test the hypothesis that in addition to VEGF, FGF-2 and hyaluronan also have critical roles in the increased neovascularization induced by mutant TIMP3 in Sorsby’s fundus dystrophy.

## 2. Materials and Methods

### 2.1. Human Samples

Patients with AMD and controls (without AMD or any other retinal disease) were recruited from the eye clinics at Cole Eye Institute under Cleveland Clinic Foundation approved IRB protocols. Plasma samples were prepared and stored at −80 °C. Samples from patients (n = 49, with 26 males and 23 females) given a clinical diagnosis of geographic atrophy or CNV and age-matched controls (n = 59 with 28 males and 31 females) were included in this pilot study to evaluate HA in the plasma. Normal and/or AMD post-mortem eyes were obtained from the Cleveland Eye Bank, the National Disease Research Interchange (Philadelphia, PA, USA) or from the Cole Eye Institute Eye Tissue Repository through the Foundation Fighting Blindness (FFB) Eye Donor Program (Columbia, MD, USA). All post-mortem tissue were obtained in accordance with the policies of the Eye Bank Association of America and the Institutional Review Board of the Cleveland Clinic Foundation (IRB#14-057). Eye bank records accompanying the donor eyes indicated whether the donor had AMD or no known eye diseases. The analyzed tissue included FFB donations #714 (82 y.o.), #781 (80 y.o.), #711 (83 y.o.), #722 (90 y.o.), #716 (80 y.o.) and #739 (90 y.o.), identified as AMD. Postmortem eyes from a 95 (#784), 92 (#979) and a 91 year-old donor without a history of retinal disease were used as controls. Eyes were enucleated 4 to 22 h postmortem and fixed in 4% paraformaldehyde and 0.5% glutaraldehyde in phosphate buffer. The globes were stored in 2% paraformaldehyde in D-PBS.

### 2.2. Mice

All mice utilized in this study were housed in the Cole Eye Institute vivarium under approved Institutional Animal Care and Use Committee (IACUC) protocols. All procedures on the mice were in accordance with ARVO statement for the Use of Animals in Ophthalmic and Vision Research and conformed to the National Institutes of Health Guide for the Care and Use of Animals in Research and to the ARVO statement for the use of animals in ophthalmic and vision research. Timp3^+/S179C^ mice were generated in the laboratory of Dr. Bernhard Weber using site-directed mutagenesis and homologous recombination in embryonic stem (ES) cells to generate mutant ES cells carrying the *Timp3^S179C^* allele. Heterozygous breeding of Timp3^+/S179C^ [33] produced homozygous Timp3^S179C/S179C^ mice and age-matched littermate controls in a C57BL6 background. Similarly, heterozygous Timp3^+/-^ mice [34] were bred to generate Timp3^-/-^ knockouts and TIMP3^+/+^ littermate controls. Eyes were enucleated following euthanasia and fresh frozen in tissue-plus optical cutting temperature embedding medium (Scigen, #4583) for sectioning and histology. Blood samples were collected via cardiac puncture and plasma prepared via standard protocols.

### 2.3. Hyaluronan Enzyme-Linked Immunosorbent Assay (ELISA)

Plasma from human patients with and without AMD and from SFD mouse models were measured for HA contents by solid-phase sandwich ELISA in 96-well plates (Costar, #9018) using the Hyaluronan Duo-Set ELISA kit (R&D Systems, #DY3614-05).

### 2.4. Immunofluorescence

Retina sections and flat-mounted ARPE-19 cells grown on polyester trans-wells were fixed for 5 min in 4% paraformaldehyde and blocked in 1% bovine serum albumin with 0.1% Triton X-100 in phosphate-buffered saline. Human sections were processed with melanin bleaching kit to remove autofluorescence (Polysciences, Inc., Warrington, PA, USA, #24883A-B). Samples were incubated overnight with biotinylated HA binding protein, (Millipore Sigma, #385911) or primary antibodies (anti-ezrin, clone 3C12, Invitrogen, Carlsbad, CA, USA #MA5-13862) in humidified chambers at 4 °C. Subsequently, secondary antibodies (anti-mouse AlexaFluor 594, streptavidin-AlexaFluor 488, streptavidin-AlexaFluor 647, all from ThermoFisher Scientific, Waltham, MA, USA) were incubated with samples at room temperature for one hour in the dark. Rhodamine-phalloidin (Thermo Fisher Scientific, R415) was incubated together with secondary antibodies. Then, 4′,6-diamidino-2-phenylindole (DAPI) was used to stain nuclei of murine sections and cell culture mounts and SYTOX green (ThermoFisher Scientific, #S7020) was used to stain nuclei in human sections. Imaging by confocal microscopy was performed (Leica TCS-SP8, Exton, PA, USA). The localization of Bruch’s membrane was determined by its autofluorescence at 405 nm.

### 2.5. Hyaluronidase Treatment of Retina Sections

Hyaluronidase from *Streptomyces hyalurolyticus* (Millipore Sigma, Burlington, MA USA, #H1136) was used to treat retina sections as described previously [35]. Streptomyces hyaluronidase was resuspended in 0.1 M sodium acetate buffer, pH 5.0, at 100 U/mL. To prevent any nonspecific digestion, the following protease inhibitors were added to the sodium acetate buffer: 1 mM iodoacetic acid, 1 mM phenylmethyl sulfonylfluoride, 1 mM EDTA, 1 μg/mL pepstatin A, 250 μg/mL ovomucoid. Hyaluronidase solution (100 mU/mL of hyaluronidase in PBS with CaCl_2_ (0.1 g/L) and MgCl_2_ (0.1 g/L)) was applied onto the sections for 3 h at 37 °C. Slides were subsequently fixed in 4% paraformaldehyde and examined by fluorescence microscopy.

### 2.6. Cells and Reagents

ARPE-19 cells stably expressing S179C-TIMP3, wild-type-TIMP3 (WT), or vector alone were reported previously [19]. Cells were expanded in DMEM-F12 with 10% FBS before transfer to polyester inserts coated with mouse laminin (Corning Inc., Corning, NY, USA, #23017). 720,000 cells and 100,000 cells were plated per well in each well of a 12-well plate or 24-well plate, respectively using a previously published protocol [36]. Essentially, ARPE-19 cells were cultured for at least 2 weeks in nicotinamide-supplemented media with 1% FBS. Media were replaced twice per week. Cells were serum-starved for 24 h before treatment with the FGF Receptor inhibitor BGJ-398 (Selleckchem, Houston, TX, USA, #S2183) for 48 h. Similarly, cells were treated with FGF-2 (Gibco from Thermo Fisher Scientific, #13256-029) with the required cofactor heparin sodium salt (1 μg/mL, Sigma Aldrich, #H3149) for 48 h after serum starving for 24 h.

### 2.7. Quantitation of Immunofluorescence by Integrated Density Analysis

Fluorescence intensity of HABP staining was quantified using integrated density analysis as previously described [37,38]. For all the RPE cell culture confocal microscopy images, fluorescence was quantitated using a standard measure of integrated density, which is the product of area and mean gray value. A custom written automated image analysis code was developed using Matlab (MATLAB 2019a, The MathWorks, Inc., Natick, MA, USA) for separating the desired color channel from the image, thereby obtaining the total area (in pixels), the mean gray value, and the integrated density.

### 2.8. In Vivo Imaging and Laser Injury Model

Laser mediated CNV was induced as described previously [28]. Briefly, mice were anesthetized with 65–68 mg/kg sodium pentobarbital delivered intra-peritoneally. Topical 0.5% procaine solution was applied for cornea anesthesia. Following anesthesia, pupils were dilated with 0.5% topical tropicamide/phenylephrine combination drops (Santen Pharmaceuticals, Osaka, Japan).

Four laser spots were placed in the superior, superior-temporal, or superior-nasal quadrants of the fundus using a green solid-state laser (Oculight by Iridex Corp., Mountain View, CA, USA) (532 nm; 2500 mW; 0.50 s pulse duration; 50 μm spot size) using a slit lamp delivery system and a microscope coverslip placed and affixed to the cornea with a drop of Systane Ultra artificial tears (Alcon, Ft Worth, TX, USA). All animals were scanned immediately after laser injury with optical coherence tomography (Envisu R2210 UHR Leica Microsystems Inc., Wetzlar, Germany) to confirm successful RPE-Bruch’s membrane rupture, an endpoint in laser-induced CNV models.

### 2.9. Statistical Analysis

**Figure 1:**
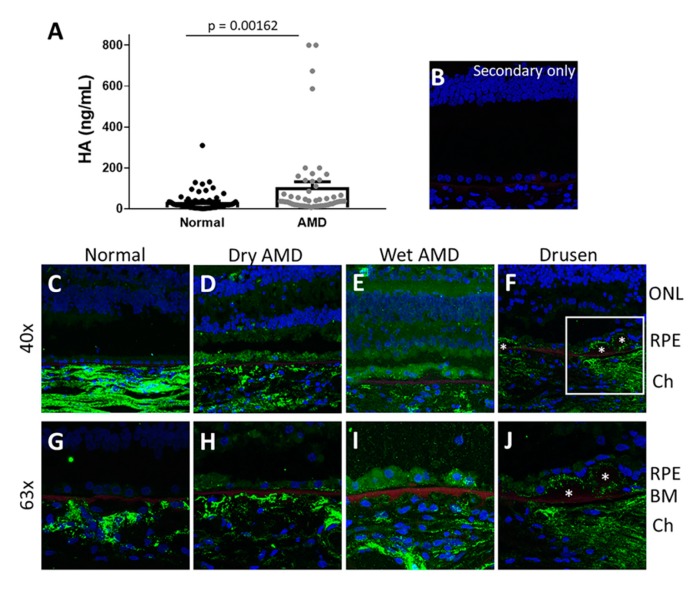
Hyaluronan HA is increased in circulation and in the RPE of age-related macular degeneration (AMD) patients. (**A**) HA was increased in plasma from patients with late-stage AMD (GA or CNV) compared to age-matched, normal controls. Data are presented as mean ± SEM (**B**–**J**) Representative human retina sections stained with HA binding protein (HABP) (**B**) Human retina section stained with streptavidin-AlexaFluor 647 in the absence of HA binding protein serves as a specificity control. (**C**–**J**) Human retina sections stained with streptavidin-AlexaFluor 647 in the presence of HA binding protein. HA is increased in the RPE in patients with dry AMD (**D**,**H**), wet AMD (**E**,**I**), and around drusen (**F**,**J**) compared to the RPE from an aged-match normal control (**C**,**G**). 40× images (**B**–**F**), 63× images (**G**–**J**). Green: HA; red: Bruch’s membrane determined by its autofluorescence at 405 nm; blue: DAPI. Asterisks indicate drusen (**F**,**J**). GA—geographic atrophy; CNV—choroidal neovascularization; ONL—outer nuclear layer; RPE—retinal pigment epithelium; Ch—choroid; DAPI—4′,6-diamidino-2-phenylindole.

**Figure 2:**
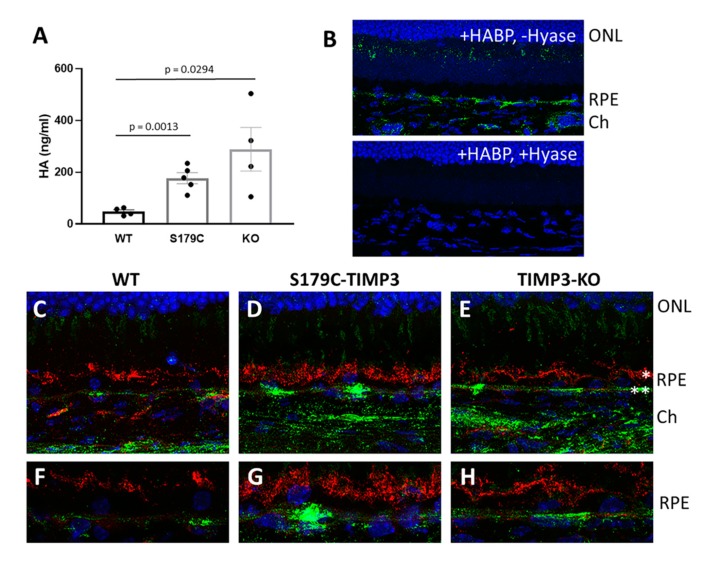
HA is increased in circulation and in the RPE and choroid in mouse models of Sorsby’s fundus dystrophy. (**A**) HA was increased in plasma from S179C-TIMP3 knockin mice (Timp3^S179C6/S179C^) and TIMP3-KO (Timp3^-/-^) mice compared to wild-type (WT) littermates. (n ≥ 5). Data are presented as mean ± SEM. (**B**) Mouse retina sections stained with biotinylated HA binding protein (HABP) in the absence (upper panel) or presence (lower panel) of hyaluronidase to detect HA. HABP staining is specific for HA as shown by the absence of staining in sections treated with hyaluronidase (lower panel). Green: HA; blue: DAPI. (**C**–**H**) Representative images of HA staining of mouse sections from wild-type (WT) mice (**C**,**F**), S179C-TIMP3 mutant mice (**D**,**G**) and TIMP3-KO mice (**E**,**H**). HA is increased in the RPE and choroid of S179C-TIMP3 (**D**) and TIMP3-KO (**E**) mice compared to wild-type (WT) littermate controls (**C**). HA (green) is predominantly localized to the basal surface of the RPE (**) (**F**–**H**) and not to the apical surface (*) as shown by co-staining with ezrin, a marker for the apical microvilli of RPE (red). 40× images (**C**–**E**); 63× images (**F**–**H**). n ≥ 3 for all immunohistochemistry data.

**Figure 3:**
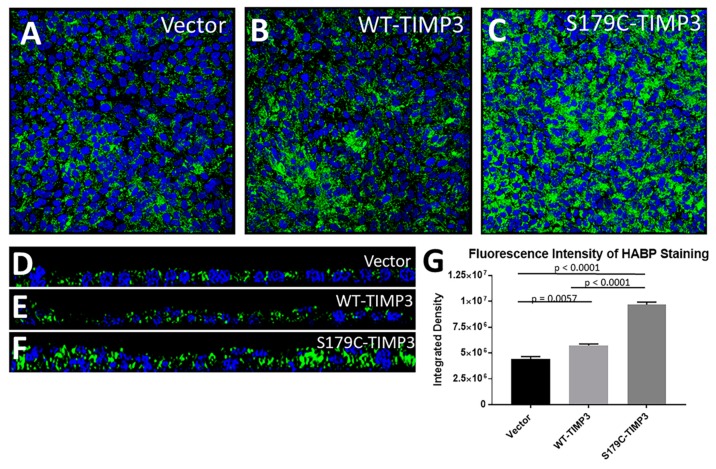
HA is increased in S179C-TIMP3 RPE cells in culture. (**A**–**C**) ARPE-19 cells expressing S179C- TIMP3 grown in culture for at least 2 weeks on trans-well inserts have increased HA (**C**) compared to WT-TIMP3 expressing cells (**B**) or vector only controls (**A**).(**D**–**F**) Z-plane images of HA in RPE monolayers grown on trans-well inserts show increased intracellular HA in S179C-TIMP3 cells. Green: HA; blue: DAPI. (**G**) Fluorescence intensity was quantitated by integrated density measurement (n ≥ 4, for each cell line). Data are presented as mean ± SEM.

**Figure 4:**
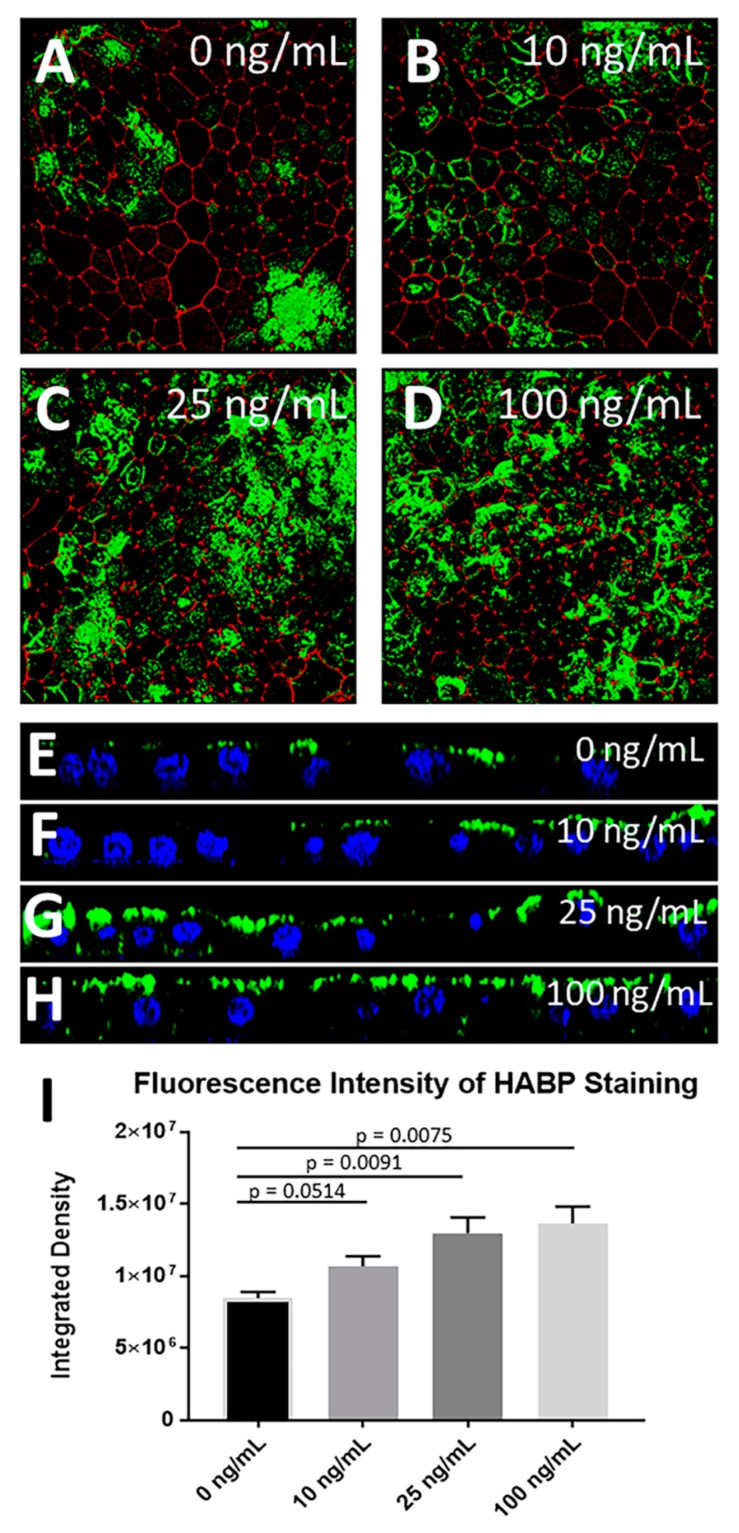
FGF-2 induces HA accumulation in primary RPE cells. (**A**–**D**) FGF-2 induced HA accumulation in primary porcine RPE cells in a dose-dependent manner (**A**) 0 ng/mL, (**B**) 10 ng/mL, (**C**) 25 ng/mL, (**D**) 100 ng/mL. Fluorescence intensity was quantitated by integrated density measurement (**I**) (n ≥ 4, for each cell line). Data are presented as mean ± SEM (**E**–**H**) Z-plane images show that increased concentrations of FGF-2 induce increased apical accumulation of HA in addition to some peri-cellular and basal deposits of HA at high doses of FGF-2. Green: HA, red: phalloidin; blue: DAPI.

**Figure 5:**
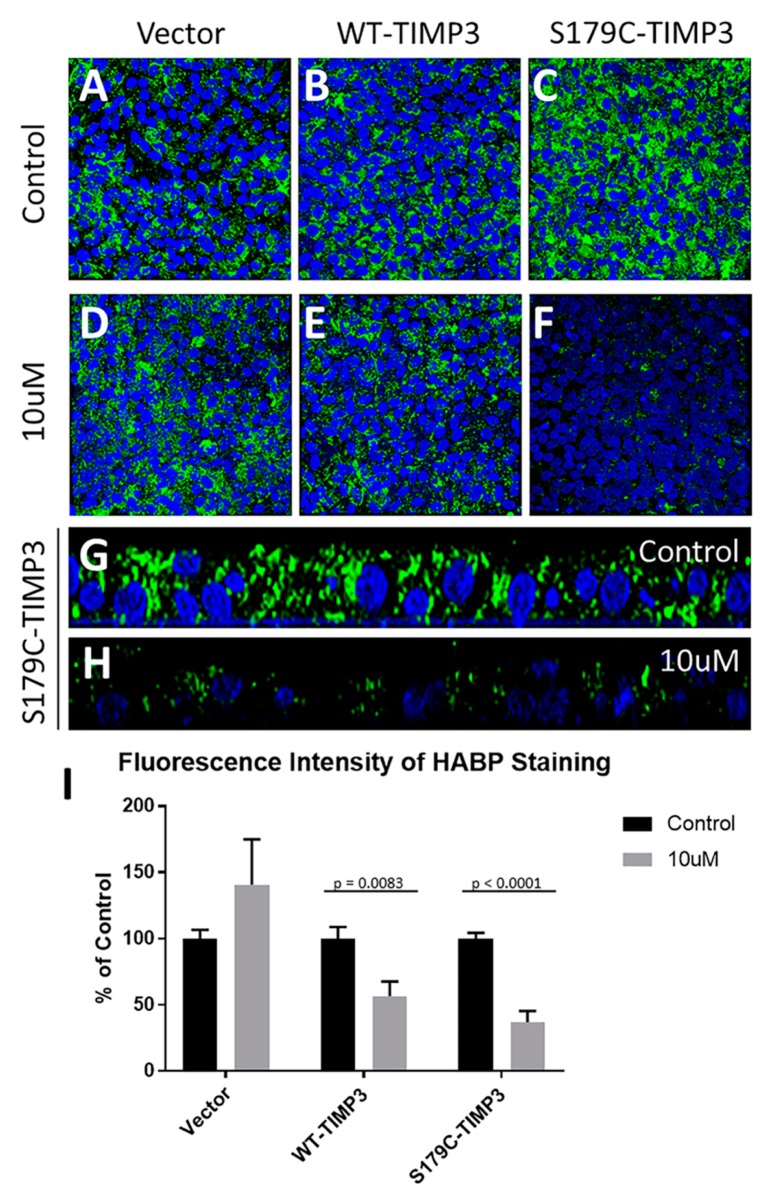
Inhibition of FGF signaling decreases HA accumulation in S179C-TIMP3 RPE cells. Vector only (**A**), WT-TIMP3 (**B**), and S179C-TIMP3 (**C**) expressing ARPE-19 cells were grown on trans-well inserts for 4 weeks before treatment with FGF receptor inhibitor BGJ-398. Treatment with BGJ-398 decreased HA accumulation in S179C-TIMP3 expressing RPE cells (**F**,**H**,**I**) and to a lesser extent in WT-TIMP3 expressing cells (**E**,**I**) but has no effect on control vector RPE cells (**A**,**D**,**I**). (**I**) Control (Black Bar) indicates respective untreated cells compared with BGJ-398 treated cells (Grey bar). (**G**,**H**) Z-plane images of S179C-TIMP3 cells in the absence (**G**) and presence (**H**) of 10 μM BGJ-398 S179C-TIMP3 cells show an overall reduction in HA accumulation, including intracellular HA, after treatment with BGJ-398. Green: HA; blue: DAPI. (**I**) data are presented as mean ± SEM (n ≥ 6).

All parameters in the study were distributed normally. Data are expressed as mean ± SEM. Differences were tested by unpaired t-test (Figure 1, Figure 2 and Figure 3) or by using multiple t-tests employing two-stage linear step-up procedure of Benjamini, Krieger and Yekutieli, with a false discovery rate set to 1% (Figure 4 and Figure 5). Each group was analyzed separately without the assumption of consistent standard deviation. *p* < 0.05 values were considered statistically significant. Statistical analysis was performed with GraphPad Prism 7.03 (GraphPad Software, Inc., San Diego, CA, USA).

## 3. Results

### 3.1. Hyaluronan is Elevated in Plasma and RPE/Choroid of Patients with AMD

Age-related macular degeneration (AMD) is usually seen as two main types. “Dry” AMD where deposits called drusen develop in the macular region that ultimately progress to a late stage in which there is atrophy of the macula (geographic atrophy). “Wet” AMD describes AMD in which patients develop abnormal growth and leakage of the choroid vessels beneath and into the retina, termed choroidal neovascularization (CNV). HA contents were measured in plasma from patients with late stage AMD (geographic atrophy or choroidal neovascularization) and from age-matched controls without the disease. ELISA analysis (Figure 1A) indicates that HA contents were significantly increased in the plasma of patients with late-stage AMD (mean ± SEM: 111.8 ± 5.78 ng/mL) compared with plasma of controls without AMD (32.91 ± 5.75).

To evaluate the distribution of HA in the retina under physiological and pathological conditions, sections from post-mortem human donor eyes from 3 controls and 6 AMD (4 dry and 2 wet AMD) patients were stained for HA using biotinylated HA binding protein (HABP). HA was found to be localized predominantly in the choroid of normal eyes (Figure 1C) as described previously [39,40]. Increased deposition of HA was seen around the RPE in AMD eyes (both in the dry (Figure 1D,H) and wet AMD specimens (Figure 1E,I)). HA was particularly enhanced in drusen and in areas of atrophy (Figure 1F,J) in AMD specimens. The sections stained with secondary antibody alone serves as a specificity control and shows minimal staining compared with sections stained with HABP (Figure 1B).

### 3.2. Increased Plasma HA and Accumulation of HA in the RPE of SFD Mice

We utilized two mouse models to study the potential role of TIMP3 in the regulation of HA in the retina: mice lacking TIMP3 [34] and mice carrying the S179C-TIMP3 SFD mutation [33]. Plasma from S179C-TIMP3 and TIMP3-KO mice at 4–6 weeks of age was collected and HA contents were analyzed by ELISA. HA content of plasma was significantly increased in mice lacking TIMP3 as well as in mice carrying the S179C-TIMP3 mutation (Figure 2A), suggesting that TIMP3 may be important in regulating HA.

To determine if there was a similar correlation between the plasma HA levels and the accumulation of HA in the RPE as observed in human sections with AMD, we evaluated accrual of HA in the retinas of mice (8 weeks of age) lacking TIMP3 or carrying the SFD mutation. Cryosections of retina from mice of each specific genotype and wild-type littermates were stained for HA content with HABP. To ascertain that HABP binds HA specifically, sections were treated with hyaluronidase prior to staining with HABP. Indeed, pre-treatment with hyaluronidase resulted in absence of staining with HABP (Figure 2B, lower panel). S179C-TIMP3 mice (Figure 2D) and TIMP3-KO mice (Figure 2E) show increased accumulation of HA beneath the RPE and in the choroid compared to that seen in wildtype littermates (Figure 2C). Staining with antibodies to ezrin served as a marker for RPE apical microvilli (Figure 2C–H), and the higher magnification images (Figure 2F–H) confirmed the RPE localization of HA to the basal surface of the cells.

To identify the potential mechanism by which S179C-TIMP3 regulates HA we utilized stable human RPE lines (ARPE-19) expressing S179C-TIMP3 [20]. ARPE-19 cells (expressing S179C-TIMP3, wildtype TIMP3 (WT-TIMP3) and empty vector (Vector)) were cultured for 2–6 weeks in 1% serum on trans-well inserts and stained for HA. Increased accumulation of HA was observed in RPE cells expressing S179C-TIMP3 (Figure 3C,G) compared with cells transfected with empty vector (Figure 3A,G) or expressing wildtype TIMP3 (Figure 3B,G). The accumulation was predominantly intracellular and apical in the RPE (Figure 3D–F). There appear to be multiple layers of S179C-TIMP3 RPE cells on the transwell compared with a single monolayer for WT-TIMP3 and vector cells, which might suggest epithelial-mesenchymal transition.

### 3.3. FGF-2 Contributes to HA Accumulation in the RPE

We have recently reported that RPE cells expressing S179C-TIMP3 secrete higher amounts of FGF-2 compared with control cells [28]. Previous studies have suggested that FGF signaling has the propensity to increase HA accumulation [32]. To experimentally test this hypothesis in RPE cells, we evaluated the ability of FGF-2 to induce HA accumulation in primary porcine RPE cells. Cells cultured for 3 weeks on trans-well inserts in 1% serum were treated with 0 ng/mL, 10 ng/mL, 25 ng/mL, or 100 ng/mL of FGF-2 in the presence of 1 μg/mL heparin, a cofactor for FGF receptor signaling. FGF-2 induced HA accumulation in a dose-dependent manner (Figure 4A–I) with maximum HA deposits being observed with a dose of 25 and 100 ng/mL (Figure 4D,H). This was confirmed by quantitation of fluorescence by integrated density measurements (Figure 4I). The FGF-2 induced accumulation of HA was seen predominantly on the apical surface of the RPE with increased basal and peri-cellular accumulation at higher doses (Figure 4E–H).

To determine if the accumulation of HA seen in RPE cells expressing S179C-TIMP3 was a consequence of increased FGF signaling, cells (RPE cells expressing S179C-TIMP3, wildtype TIMP3 or empty vector) were treated with BGJ-398, an FGF receptor inhibitor. 10 μM BGJ-398 decreased HA content in RPE cells expressing S179C-TIMP3 (Figure 5C,F,H,I) and WT-TIMP3 (Figure 5B,E,I) but not vector only transfected cells (Figure 5A,D,I) when compared with their respective untreated cells that served as controls (Figure 5A: vector, 5B: WT-TIMP3 and 5C,G: S179C-TIMP3). Quantitation of fluorescence by integrated density analysis revealed that 10 μM BGJ-398 decreased HA accumulation in S179C-TIMP3 cells 63.1% (SD = 0.241) and only 43.4% (SD = 0.291) in wildtype cells (Figure 5I). Quantitation confirmed that BGJ-398 had no significant effect on vector only cells (Figure 5I), suggesting an FGF-specific mechanism for HA accumulation in S179C-TIMP3 cells.

### 3.4. Increased HA is Associated with CNV in AMD and SFD

**Figure 6:**
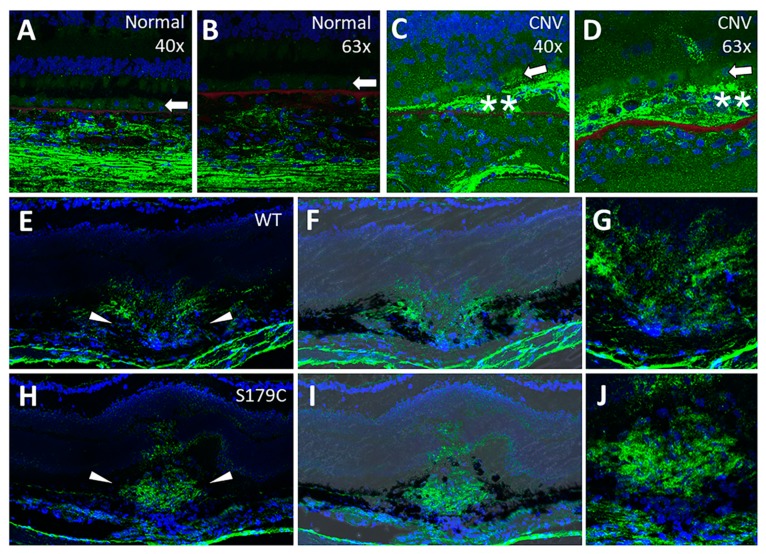
HA is increased in choroidal neovascular lesions in AMD patients and in S179C-TIMP3 mice. (**A**–**D**) HA is increased in the RPE in a patient with CNV (**C**,**D**) and in the CNV lesion compared to the RPE of a normal patient (**A**,**B**). Green: HA; blue: nuclei; red: Bruch’s membrane. Arrows indicate RPE, double asterisks indicate CNV lesion. (**E**–**G**) HA is increased in laser-induced CNV lesions in S179C-TIMP3 mice compared to wild-type (WT) littermates 5 days post injury. (**E**,**H**) Arrows indicate CNV lesion. (**F**,**I**) Brightfield image overlaid on fluorescent image shows disruption of RPE and Bruch’s membrane. (**G**,**J**) HA accumulation is diffuse and appears predominantly in the borders of the lesion in WT (**E**–**G**) compared to dense mass within the lesions of S179C-TIMP3 mice (**H**–**J**). Green: HA; blue: nuclei.

Sections of post-mortem eyes from a patient with CNV showed significant HA deposition around the RPE (Figure 6C,D) when compared with control eyes (Figure 6A,B). While S179C-TIMP3 mice do not demonstrate a florid SFD phenotype as seen in humans, they do have increased susceptibility to experimental laser-induced CNV [28] as do the TIMP3-KO mice [41]. We have previously shown that FGF-2 from S179C-TIMP3 RPE cells can stimulate angiogenesis [28]. Since FGF-2 contributes to HA accumulation in the RPE and to angiogenesis, we evaluated if HA content and distribution was altered in laser-induced CNV lesions in S179C-TIMP3 mice. As described previously lesions in S179C-TIMP3 mice were larger and leakier compared to controls [28]. Increased HA accumulation was observed in CNV in S179C-TIMP3 mice (Figure 6H–J) when compared to lesions in control mice (Figure 6E–G) which appears to be a consequence of altered distribution to the CNV lesions in S179C-TIMP3 mice.

## 4. Discussion

Sorsby’s fundus dystrophy (SFD) is a rare macular dystrophy characterized by vision loss due to persistent choroidal neovascularization [1,2,3,4,5,6]. SFD is an autosomal dominant, fully penetrant degenerative disease of the macula and is notable for its similarity in histopathological features to AMD [3,4,5,6]. The majority of SFD patients develop CNV, as well as confluent, 20–30 μm thick, amorphous deposits between the basement membrane of the RPE and Bruch’s membrane. TIMP3 and/or its downstream substrates have been postulated to have a role in the pathogenesis of both SFD and AMD, because accumulation of TIMP3 has been observed in subretinal deposits in SFD [42] as well as in AMD drusen [43,44,45]. In this study, we show that hyaluronan accumulates around the RPE in AMD as well as in CNV lesions of mice expressing S179C-TIMP3.

One interesting observation from our studies was the increase in HA in the plasma of patients with AMD as well as in mice lacking TIMP3 or carrying the S179C-TIMP3 mutation. Although we observe significant differences in the plasma levels of HA in patients with advanced AMD (GA and CNV), the number of patients analyzed (n = 49 controls) and n = 59 (AMD) is not sufficient to determine if this is of prognostic value. In addition, we did not have access to samples from patients at different degrees of severity to be able to draw conclusions with this or the rate of progression of the disease. Future studies are warranted to address this question. While patients with SFD generally demonstrate disease localized to the retina, our results may be a consequence of ubiquitous expression of TIMP3 in a variety of tissues in the body [46] which could potentially explain the systemic increase in HA. Whether there is accumulation of HA in other tissues in S179C TIMP3 mice has not been evaluated.

Our studies with RPE cells suggest that the increase in HA in these cells is likely a consequence of increased FGF. A recent study [47] suggests that TIMP proteins can control FGF-2 bioavailability in skeletal tissue and the same might be true of multiple tissues leading to systemic increase in HA in the plasma. The exact mechanism by which TIMP3 regulates FGF bioavailability in the RPE is currently unknown, but it is highly likely that the TIMP-metalloproteinase axis likely has a key role. The extracellular matrix (ECM) serves as a high capacity reservoir for FGF-2 and early studies have demonstrated that matrix metalloproteases (MMPs) have the ability to mobilize FGF-2 to a soluble phase that results in receptor activation [48]. Additional studies identifying the molecular mechanisms by which TIMP3 regulates FGF-2 bioavailability will provide insight into the pathophysiology of the disease.

FGF-2 is sufficient to increase HA accumulation and distribution in the RPE, and blocking FGF signaling in S179C-TIMP3 RPE cells brings HA levels back to normal. The mechanism by which FGF-2 increases HA accumulation is not understood. HA is endogenously synthesized by a family of membrane-integrated glycosyltransferases, called hyaluronan synthases (HAS 1-3) and is exported directly into the ECM [49,50]. Hyaluronidases (HYAL1-2) are a class of enzymes that degrade HA [51]. A balance between HA synthesizing and degrading activity keeps HA at physiological levels. In order to determine the mechanism of accumulation of HA in the RPE in SFD, we performed quantitative PCR analysis of HAS1-3 and HYAL1-2 from RPE isolated from S179C-TIMP3, TIMP3-KO mice and wildtype littermate controls. Interestingly, we observed no changes in gene expression of any of these enzymes in the mutant mice (Supplementary Figure S1). Therefore, at least in the RPE in SFD mice, the differences in HA content are not due to increased expression of the synthases nor decreased expression of canonical degradation enzymes. However, there are other possibilities that need to be explored in the future. HA production could be modulated by decreasing enzyme recycling from endosomes back to the cell surface as seen in keratocytes [52]. Additionally, there is a possibility that other non-canonical hyaluronidases such as KIAA1199 [53] and Tmem2 [54] could be involved. Alternatively, as previously reported degradation might be prevented by increased binding proteins on HA and leading to net accumulation [55].

HA has been shown to exhibit a diverse array of biological functions including a role in the response to tissue damage and inflammation [56]. Our studies demonstrating accumulation of HA in laser-induced CNV lesions corroborates previous studies [27]. This study also reported an increase in *CD44* and *HAS2* mRNA following laser injury [27]. It is possible that the increased accumulation of HA in laser-induced CNV lesions in S179C-TIMP3 mice might result from similar increases in mRNA transcription.

Chronic low-grade inflammation has been suggested to contribute to age-related macular degeneration [57]. In the laser-induced mouse model of CNV, inflammatory processes have been shown to play a role in the development and regression of the lesions. A number of reports link HA remodeling to the modulation of neuroinflammation with low-molecular weight HA being pro-inflammatory and high molecular weight HA being anti-inflammatory [58,59,60]. While we see increased deposition of HA in and around the RPE, we have not determined its physical properties such as size and molecular weight distribution in the tissue.

The receptor engagement of HA in the retina or it’s downstream signaling under physiological or pathological conditions has not yet been identified and will be important as we determine its exact role in the pathology of macular degenerative disease. In our study we demonstrate that primary porcine RPE cells deposited HA predominantly on the apical surface under physiological conditions similar to what had been previously reported for human RPE cells [61]. Our data revealed that FGF-2 induced HA accumulation apically as well as between cells and on the basal surface, suggesting that in addition to increased total HA content, the distribution of HA may be important for disease pathogenesis and warrants further investigation. We have recently reported that the secretion of FGF-2 by RPE cells expressing S179C-TIMP3 led to increased angiogenesis [28]. Whether HA is modified in the endothelial glycocalyx as a consequence of FGF-2 has not been studied and might provide further insight into the pathogenesis of CNV in AMD and SFD leading to the identification of novel therapeutic approaches.

## Supplementary Materials

The following are available online at https://www.mdpi.com/2073-4409/9/3/608/s1, Figure S1: RNA was isolated from mouse RPE using the Simultaneous RPE cell Isolation and RNA Stabilization method (SRIRS method) using the RNA Plus Mini Kit (Qiagen). Quantitative PCR was performed following reverse transcription using TaqMan probes for the mouse genes Has1 (A), Has2 (B), Has3 (No signal), Hyal1 (C), Hyal2 (D), and 18S ribosomal RNA (rRNA) (Applied Biosystems). 18S rRNA was used as endogenous control for each gene tested. mRNA expression was calculated using 2-ΔΔCt method and shown relative to expression in wildtype littermate mice.
