# Supplementary material for: Role of FGF and Hyaluronan in Choroidal Neovascularization in Sorsby Fundus Dystrophy
Source: Cells. 2020 Mar 4;9(3):608. doi: 10.3390/cells9030608 (PMC7140456; doi:10.3390/cells9030608)
Supplement: Supplementary file 1 [file cells-09-00608-s001.pdf]

Supplemental Fig. 1

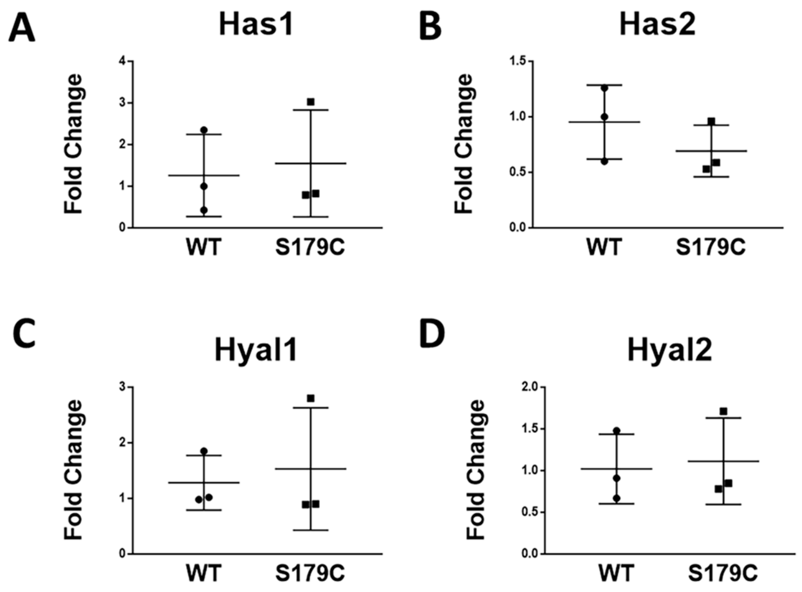

RNA was isolated from mouse RPE using the Simultaneous RPE cell Isolation and RNA Stabilization method (SRIRS method) using the RNA Plus Mini Kit (Qiagen). Quantitative PCR was performed following reverse transcription using TaqMan probes for the mouse genes Has1 (A), Has2 (B), Has3 (No signal), Hyal1 (C), Hyal2 (D), and 18S ribosomal RNA (rRNA) (Applied Biosystems). 18S rRNA was used as endogenous control for each gene tested. mRNA expression was calculated using 2-ΔΔCt method and shown relative to expression in wildtype littermate mice.
